# Glucose Metabolism Effects of Vitamin D in Prediabetes: The VitDmet Randomized Placebo-Controlled Supplementation Study

**DOI:** 10.1155/2015/672653

**Published:** 2015-05-27

**Authors:** Tomi-Pekka Tuomainen, Jyrki K. Virtanen, Sari Voutilainen, Tarja Nurmi, Jaakko Mursu, Vanessa D. F. de Mello, Ursula Schwab, Martti Hakumäki, Kari Pulkki, Matti Uusitupa

**Affiliations:** ^1^Unit of Public Health, The Institute of Public Health and Clinical Nutrition, University of Eastern Finland, P.O. Box 1627, 70211 Kuopio, Finland; ^2^The Unit of Clinical Nutrition, The Institute of Public Health and Clinical Nutrition, University of Eastern Finland, P.O. Box 1627, 70211 Kuopio, Finland; ^3^Institute of Clinical Medicine, Internal Medicine, Kuopio University Hospital (KYS), P.O. Box 100, 70029 Kuopio, Finland; ^4^Department of Clinical Chemistry, Institute of Clinical Medicine, University of Eastern Finland and Eastern Finland Laboratory Centre, P.O. Box 1627, 70211 Kuopio, Finland

## Abstract

Epidemiological evidence suggests a role for vitamin D in type 2 diabetes prevention. We investigated the effects of vitamin D_3_ supplementation on glucose metabolism and inflammation in subjects with prediabetes. A 5-month randomized, double-blind, placebo-controlled intervention with three arms (placebo, 40 *μ*g/d, or 80 *μ*g/d vitamin D_3_) was carried out among sixty-eight overweight (BMI 25–35) and aging (≥60 years) subjects from Finland, with serum 25-hydroxyvitamin D_3_ [25(OH)D_3_] < 75 nmol/L and either impaired fasting glucose or impaired glucose tolerance. Analyses included 66 subjects who completed the trial. Glucose metabolism was evaluated by fasting and 2-hour oral glucose tolerance test-derived indices and glycated hemoglobin. Inflammation was evaluated by high-sensitive C-reactive protein and five cytokines. Although a dose-dependent increase in serum 25(OH)D_3_ over the supplementation period was observed (*P* trend < 0.001), there were no other statistically significant differences in changes in the 13 glucose homeostasis indicators between the study groups other than increase in the 120 min glucose concentration (*P* trend = 0.021) and a decreasing trend both in 30 min plasma insulin (*P* trend = 0.030) and glycated hemoglobin (*P* trend = 0.024) concentrations. A borderline statistically significant decreasing trend in interleukin-1 receptor antagonist concentration was observed (*P* = 0.070). Vitamin D_3_ supplementation does not improve glucose metabolism in ageing subjects with prediabetes but may have modest anti-inflammatory effects.

## 1. Introduction

There is a lot of epidemiological evidence to suggest that vitamin D has a role in glucose homeostasis, especially in maintaining normal glucose homeostasis and protecting against type 2 diabetes [1, 2].

However, controlled supplementation trials with vitamin D or its analogues have not been able to produce similar results. In a recent systematic review and meta-analysis of 15 trials lasting from two months to 7 years, no significant improvements were seen in glucose homeostasis when all trials were included [3]. The results from the more recent trials have been inconsistent. Two trials in subjects with impaired glucose tolerance (IGT) did find a positive effect on glucose homeostasis with vitamin D_3_ supplementation [4–7], whereas three trials in subjects with IGT and one in subjects with normal glucose tolerance found no effects [8–10].

Insulin resistance, metabolic syndrome, and type 2 diabetes are associated with a systemic, chronic inflammation, where, for example, circulating levels of proinflammatory cytokines and C-reactive protein are elevated [11, 12]. Although vitamin D has immunomodulatory and anti-inflammatory properties, especially based on experimental studies [13], bodies of evidence from human studies in the prediabetic state assessing the role of moderate to large dose supplementation of vitamin D in inflammation and glucose metabolism are still relatively few.

As more evidence is clearly needed, we decided to study the placebo-controlled effect of 5-month 40 *μ*g/d (1,600 IU) or 80 *μ*g/d (3,200 IU) oral vitamin D_3_ supplementation on glucose homeostasis in an eastern Finnish population with either impaired fasting glucose (IFG) or IGT during winter time when serum vitamin D_3_ concentrations drop without supplementation. Furthermore, we assessed the anti-inflammatory effect of the supplementation by measurements of serum high-sensitive C-reactive protein (hsCRP), plasma soluble tumor necrosis factor receptor type II (psTNFrII), plasma interleukin-6 (pIL-6), plasma interleukin-1 receptor antagonist (pIL-1RA), and plasma interleukin-1 beta (pIL-1*β*) concentrations.

## 2. Materials and Methods

### 2.1. Subject Selection

The Glucose Metabolism Effects of Vitamin D Supplementation in Prediabetes (VitDmet) study was a 5-month randomized, double-blind, placebo-controlled supplementation trial that was conducted in winter 2011/2012 in eastern Finland. The primary aim was to assess the effects of vitamin D_3_ supplementation on glucose homeostasis in subjects with IFG or IGT. For inclusion the subjects needed to be ≥60 years of age with evidence of disturbed glucose homeostasis, that is, either IFG (fasting plasma glucose concentration 5.6 to 6.9 mmol/L) or IGT (oral glucose tolerance test (OGTT) 120 min plasma glucose concentration 7.8 to 11.1 mmol/L), but not type 2 diabetes (fasting plasma glucose concentration ≥ 7.0 mmol/L or OGTT 120 min plasma glucose concentration ≥ 11.1 mmol/L), and to be overweight (BMI > 25), but not severely obese (BMI < 35). Furthermore, subjects needed to have their serum 25(OH)D_3_ concentration <75 nmol/L. Exclusion criteria were any disease that could be affected by vitamin D, such as sarcoidosis, or a condition that could affect vitamin D metabolism, such as kidney disease.

**Figure 1:**
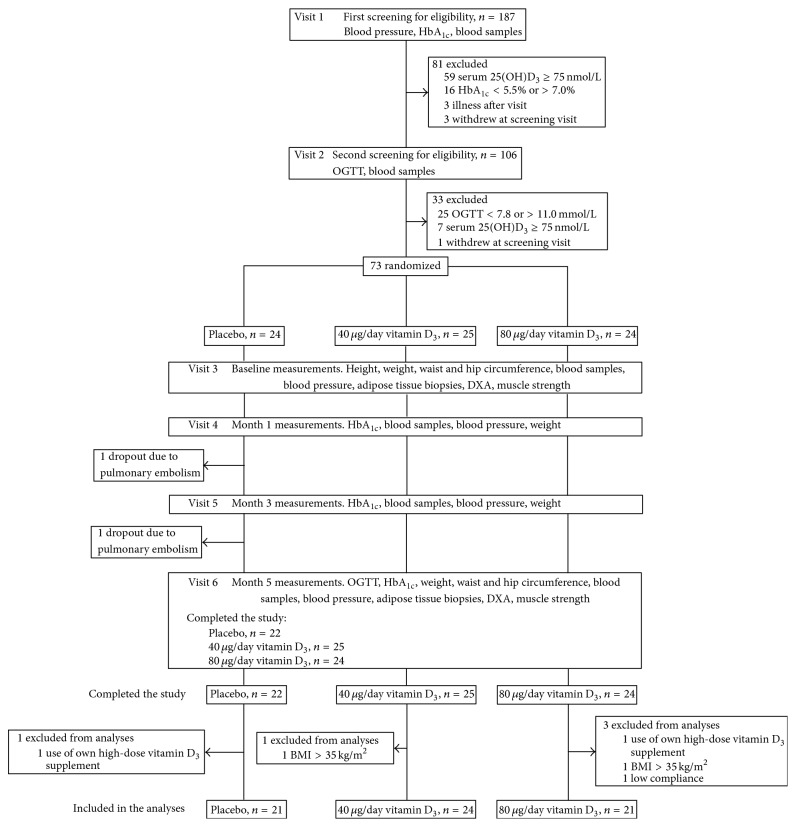
Flow diagram of the study. 25(OH)D_3_: 25-hydroxyvitamin D_3_; BMI: body mass index; OGTT: oral glucose tolerance test.

A two-stage screening was used, as shown in Figure 1. The adult voluntary subjects were recruited by advertisements in regional newspapers and after a prescreening on the phone for inclusion and exclusion criteria (self-reported elevated blood glucose, age, height, weight, and diseases) were invited for the first screening at the study clinic (visit 1). A written informed consent was collected, HbA_1c_ was measured with a point-of-care device, the subjects were interviewed, and their self-administered questionnaires were checked. If the subject's HbA_1c_ was 5.5–7.0%, fasting venous blood samples were drawn and analyzed for plasma glucose and serum 25(OH)D_3_ concentrations. Those fulfilling the inclusion criteria were scheduled for the OGTT (visit 2), after which those fulfilling all criteria were randomized to receive either placebo, 40 *μ*g/d, or 80 *μ*g/d vitamin D_3_ daily. Each dose comprised a combination of placebo and 20 *μ*g tablets, two in the morning and two in the evening. Randomization was carried out by a statistician, in the order of entry after visit 2. Study nurse distributed the containers according to the statistician-generated list and neither the nurse nor a participant knew the allocation group. All tablets and containers were physically identical, the allocation arm code hidden in the serial number on the container label. The average time between visit 2 and the beginning of the supplementation was approximately one week. During the 5-month supplementation period between October 2011 and April 2012, the subjects visited the study site three times: 1 and 3 months after the start of the supplementation and at the end of the study (Figure 1).

We aimed to recruit 102 participants, 34 into each of the three supplementation groups, but due to lower than expected public interest to participate and generally higher than anticipated serum 25(OH)D_3_ concentrations in the base population, at closing of the recruitment window we had 73 randomized subjects in the three supplementation arms (63 men and 10 women). According to power computations, to reach 80% power (i.e., *β* = 0.20) at *α* = 0.05, with a difference of 1 SD to 0.75 SD in the effect measure between the groups, there needed to be 16 to 28 subjects per group. 1 SD equals an effect size of 10% to 50% in most of the outcome parameters.

The Research Ethics Committee of the Northern Savo Hospital District has approved the study protocol. All subjects gave a written informed consent to participate in the study. The study is registered in the ClinicalTrials.gov (identification code: NCT01479933).

### 2.2. Measurements

Serum 25(OH)D_3_ concentration was measured from venous blood samples by a high performance liquid chromatography with coulometric electrode array (HPLC-CEAD), as described [14]. The interassay CV% of the method is <8.0% and the intra-assay CV% is <7.3%. Plasma glucose concentration was assayed by the photometric hexokinase method (KoneLab 20XT, Thermo Fischer Scientific, Vantaa, Finland) and plasma insulin concentration by the chemiluminescence assay (DiaSorin Liaison, DiaSorin, Dietzenbach, Germany). A two-hour OGTT was carried out with a 75-gram glucose load. Plasma glucose and plasma insulin concentrations were measured at three time points: at start (i.e., fasting), at 30 min, and at 120 min. Homeostatic modeling assessment (HOMA2) indices were computed according to the nonlinear function presented by Wallace and coworkers [15]. The insulinogenic index (IGI) was computed as follows: IGI = (30 min insulin − 0 min insulin)/(30 min glucose − 0 min glucose) in the OGTT, as described by Phillips and coworkers [16], and the insulin sensitivity index (ISI) was computed as follows: ISI = 10000/Sqr[(fasting glucose × fasting insulin) × (mean glucose × mean insulin)], as described by Matsuda and DeFronzo [17]. HbA_1c_ was measured with a point-of-care device from a capillary blood sample as instructed by the manufacturer (DCA Vantage, Siemens Healthcare Diagnostics, Deerfield, Illinois, USA). Serum liver enzymes gamma-glutamyl transpeptidase (sGGT) and alanine aminotransferase (sALAT) were determined with enzymatic photometric IFCC methods, kidney function marker serum creatinine (sCREA) with an enzymatic photometric test, and serum total calcium (sCA) with colorimetric Arsenazo III test at site with a clinical chemistry analyzer (Konelab 20XT, Thermo Fisher Scientific, Vantaa, Finland). Serum parathyroid hormone (sPTH) and serum thyroid stimulating hormone (sTSH) were assayed by chemiluminescence methods (DiaSorin Liaison, DiaSorin, Dietzenbach, Germany). Total blood count (TBC) and inflammatory cytokines hsCRP, psTNFrII, pIL-6, pIL-1RA, and pIL-1*β* were measured in Eastern Finland Laboratory Centre, Kuopio, Finland. HsCRP was measured with Roche Cobas 6000 automated analyser. Plasma IL-6, IL-1RA, soluble TNFRII, and IL-1*β* were measured with ELISA from R&D Systems (Minneapolis, MN, USA).

Body weight was measured to 0.1 kg with subjects wearing light underwear, with a recently calibrated Vetek TI-1200 scale (Vetek, Sweden), and height in a Frankfurt position to 0.5 cm by a wall-mounted device (KaWe, Germany). Body mass index (BMI) was computed as weight/height squared (kg/m^2^). Blood pressure was measured after a 5 min rest from the upper arm at a supine position, using a recently calibrated semiautomatic device (OMRON Intellisense M7, OMRON Healthcare, Netherlands). Systolic and diastolic blood pressure values were computed as the mean of the last two of three measurements.

Participant compliance in taking supplements as instructed was assessed by counting the study supplements not consumed at the last study visit.

### 2.3. Statistical Analysis

From the statistical analyses we excluded two subjects who had an increase in their BMI to >35 kg/m^2^ between the randomization and the start of the supplementation (visit 3), two subjects who reported at the end of the study that they had started to use a high-dose (50 *μ*g/day and 125 *μ*g/day) vitamin D_3_ supplement in addition to the study supplements during the study period, and one subject due to low compliance. The subject with the low compliance in the 80 *μ*g/day group had used only 17% of the study tablets. After the exclusions, the number of subjects in the analyses with the baseline data was 68 (Table 1) and in the analyses with the change variables 66 (Table 2), after the two dropouts (Figure 1) were excluded.

Glucose homeostasis was assessed by fasting glucose, insulin, and HbA_1c_ measurements and OGTT glucose and insulin measurements. The derived variables to be tested were the change in the chosen variables over the supplementation period. These were as follows: serum HbA_1c_ (concentration and %), fasting, 30 min and 120 min glucose and insulin concentrations, HOMA2 insulin resistance (HOMA2-IR), insulin sensitivity (HOMA2-IS) and beta cell function (HOMA2-B%), IGI, and ISI. Inflammatory effects were assessed by analyzing the change in the hsCRP, psTNFrII, pIL-6, pIL-1RA, and pIL-1*β* concentrations over the supplementation period. Physiological and safety indicators were changes in the 25(OH)D_3_, sPTH, sCA, sCREA, sGGT, sALAT, and sTSH.

As a large proportion of the variables did not follow the normal distribution, we used the Kruskal-Wallis test (*P*_K-W_) to test the intergroup differences, Mann-Whitney *U* test (*P*_M-WU_) to test the pairwise intergroup differences (placebo versus 40 *μ*g/d and placebo versus 80 *μ*g/d), and the Jonckheere-Terpstra test (*P*_J-T_) for trend across the groups. Related-Samples Wilcoxon Signed Rank test (*P*_WSR_) was used to study the intragroup change over time. A two-sided *P* < 0.05 was considered statistically significant for the Kruskal-Wallis test, a 1-sided *P* < 0.05 for the Jonckheere-Terpstra test, and a two-sided *P* < 0.025 for the Mann-Whitney *U* test (i.e., with the Bonferroni correction). All statistical analyses were computed with the SPSS Statistical Package for Windows, version 21.0 (Armonk, NY: IBM Corp.).

The supplementation effect analyses were conducted* per protocol;* as for the dropouts there were no data available at the end of the study to compute the change variables.

## 3. Results

### 3.1. Randomization

Despite the moderately small sample size, the randomization yielded reasonably identical groups with regard to vitamin D and glucose metabolism-related parameters.

### 3.2. Subject Characteristics

Characteristics of the 68 study subjects at baseline are presented in Table 1. The median age of the subjects at entry was 65.7 (interquartile range 62.7 to 69.7) years, and the median serum 25(OH)D_3_ concentration was 57.2 (interquartile range 47.2 to 67.2) nmol/L. The arithmetic mean 25(OH)D_3_ concentration was 57.0 nmol/L with a standard deviation of 11.0 nmol/L. One of the 68 subjects had serum 25(OH)D_3_ concentration under 30 nmol/L and 18 subjects had concentration below 50 nmol/L.

Of the 68 subjects that entered the supplementation period, 66 completed the study (Figure 1).

### 3.3. Serum 25(OH)D_3_ and PTH

There was a marked dose-dependent increase in the average serum 25(OH)D_3_ concentration in the three study groups, from 55.3 to 59.0 nmol/L, from 57.7 to 85.4 nmol/L, and from 58.1 to 103.1 nmol/L, in the placebo, 40 *μ*g/d, and 80 *μ*g/d groups, respectively (mean ranks for change 17.1, 35.4, and 47.8, resp., *P*_K-W_ < 0.001; see Table 2). At baseline, one of the subjects in the 80 *μ*g/day group had serum 25(OH)D_3_ >75 nmol/L. At the end of the study, 3/21 (14%) subjects in the placebo group, 16/24 (67%) subjects in the 40 *μ*g/d group, and 18/21 (86%) subjects in the 80 *μ*g/d group had serum 25(OH)D_3_ > 75 nmol/L. Concomitant with the increase in the serum 25(OH)D_3_ concentrations, there was a dose-dependent decrease in the serum PTH concentrations (Table 2).

### 3.4. Glucose Metabolism

Changes in the tested glucose homeostasis parameters over the supplementation period are given in Table 2. The only statistically significant effect between the groups was an increase in the 120 min plasma glucose concentration, that is, opposite to expected (*P*_K-W_ = 0.039, *P*_J-T_ = 0.021), although only the pairwise group difference for the placebo versus 40 *μ*g/d was statistically significant after Bonferroni correction (*P* = 0.022), and a decreasing trend in the HbA_1c_ concentration (*P*_J-T_ = 0.024) and in the 30 min insulin concentration (*P*_J-T_ = 0.030). Borderline statistically significant trend was observed in the IGI (*P*_J-T_ = 0.063).

### 3.5. Inflammation

In the tested inflammation markers, shown in Table 2, the only borderline statistically significant finding was a decreasing trend in the plasma IL-1RA concentration (*P*_J-T_ = 0.070). There were detectable concentrations of pIL-1*β* only for four subjects at entry and for two subjects at the end of the study and therefore the *P* values cannot be considered reliable.

### 3.6. Safety

No statistically significant changes were observed in sCA (Table 2) or in the parameters of liver or kidney function or in the TBC (data not shown). No statistically significant differences between groups were observed in changes in waist circumference or BMI, in blood pressure, or in circulating lipids (data not shown).

### 3.7. Sensitivity Analyses

We repeated the analyses by including also those with BMI > 35 at the start of the supplementation period, those who started their own high-dose vitamin D supplementation during the trial, or the one with low compliance (*n* = 71). The results were generally similar to those with the exclusions, except for the *P* for trend for IL1-Ra, which was statistically significant (*P*_J-T_ = 0.027).

## 4. Discussion

Our study showed that oral supplementation with daily 40 *μ*g or 80 *μ*g doses of vitamin D_3_ for five months markedly increases the circulating concentrations of 25(OH)D_3_, even over the winter months that usually are associated with a decrease in the 25(OH)D_3_ in this population [18]. We also showed that the 80 *μ*g dose is more potent than the 40 *μ*g dose in increasing serum 25(OH)D_3_ and that a considerably large proportion of the subjects in both supplementation groups reached serum 25(OH)D_3_ concentrations above 75 nmol/L. Furthermore, we showed that the supplementation also resulted in a decrease in serum PTH concentration but did not induce a rise in serum total calcium concentration. Vitamin D_3_ supplementation, even at 80 *μ*g per day, was also well tolerated and safe.

The primary outcome of the study, body glucose homeostasis, remained for the most part practically unchanged, thus speaking against the role of vitamin D_3_ as an important regulator of glucose homeostasis in an ageing Finnish population with prediabetes. Among the 13 glucose homeostasis indices analyzed, the 120 min plasma glucose was the only index that was statistically significant also in the pairwise analyses. We believe that other changes observed (30 min insulin, HbA_1c_) may be related to random fluctuation of glucose metabolism in individuals with impaired glucose metabolism. Among the interventional studies on glucose homeostasis indicators in populations with prediabetes, an open-label study by Nazarian et al. showed an improvement in insulin sensitivity in 11 IFG subjects after a 4-week 10,000 IU/d (250 *μ*g/d) vitamin D_3_ intervention [4]. In an earlier* post hoc* analysis by Pittas et al. among the 92 subjects with IFG at baseline [19], there was a lower rise in fasting plasma glucose concentration and lower increase in HOMA-IR in 3 years in the group taking 700 IU (17.5 *μ*g) vitamin D_3_ plus 500 mg calcium citrate daily as compared with subjects taking placebo. Glucose homeostasis was also improved in the study by Mitri et al., where 16-week vitamin D_3_ supplementation of 2,000 IU/d (50 *μ*g/d) improved *β*-cell function and attenuated the rise in HbA_1c_ [5]. In an open-label study by Dutta et al. in subjects with IGT or IFG, vitamin D_3_ supplementation of 60,000 IU (1500 *μ*g) once per week for 8 weeks and then monthly along with 1250 mg of calcium carbonate/d resulted in lower fasting blood glucose and improvement in insulin resistance during an average follow-up of 28 months, when compared with subjects taking only calcium carbonate [6]. In a recent placebo-controlled study by Gagnon et al., vitamin D_3_ upplementation for six months with a dose aimed at increasing the serum 25(OH)D_3_ to ≥75 nmol/L (2000–6000 IU/d, 50–150 *μ*g/d) improved insulin sensitivity in subjects with prediabetes, but not in subjects without prediabetes [7]. In contrast, in a study by Harris et al. in 89 overweight or obese African American subjects, 12-week supplementation with 4,000 IU/d (100 *μ*g/d) of vitamin D_3_, compared with placebo, led to reduced insulin sensitivity and increased insulin secretion [8]. In the 12-month supplementation trial by Davidson et al. in 117 Latino and African American subjects in California with hypovitaminosis D_3_ (i.e., serum 25(OH)D_3_ concentration <30 ng/mL (75 nmol/L)), the average weekly dose of 88,865 IU (2,222 *μ*g, or about 317 *μ*g/day) of vitamin D_3_ had no effect on insulin secretion, insulin sensitivity, or development of type 2 diabetes, as compared with placebo [9]. Our results support these null findings on glucose homeostasis in Finns, a Caucasian population at northern latitudes, even though we have earlier observed vitamin D_3_ sufficiency to associate with the lower risk of type 2 diabetes in an observational study of the same source population [20]. The observed increase in the 120 min plasma glucose is somewhat unexpected, but one explanation may simply be a chance finding due to a largish number of tests that were carried out. The increase was larger in the 40 *μ*g group than in the 80 *μ*g group, which supports that the finding occurred by chance as we believe is the case with other variables we monitored, that is, 30 min insulin and HbA_1c_.

Taken together, the evidence from the observational studies supports the association between low vitamin D status and impaired glucose metabolism, but the results from the experimental studies have not found improvements in glucose metabolism with vitamin D supplementation. A recent Mendelian randomisation study that looked into the association between body vitamin D status and glucose homeostasis and type 2 diabetes with four SNPs and four glycaemic traits showed an association in two SNPs near genes related to 25(OH)D synthesis with fasting insulin, but not in any of the other eleven tests conducted [21]. However, if a limitation is to be pointed out, many of the trials have been conducted in subjects with healthy glucose homeostasis or in subjects with diabetes, and only few have been carried out in subjects with prediabetes, the subjects that would probably show any effect on glucose homeostasis most easily. Therefore, well-designed randomized trials with large sample sizes and in people with insufficient vitamin D status may be needed to elucidate the role of vitamin D supplementation in glucose homeostasis and in prevention of type 2 diabetes.

We found supplemental vitamin D_3_ to exert some anti-inflammatory action, when a borderline statistically significant decrease in the IL-1RA concentration was found. IL-1RA is regarded as one of the most sensitive markers of inflammation, as it is readily secreted by many cell types and plasma levels are high enough to be reliably measured. Elevated values of IL-1RA have also been shown to predict the onset of type 2 diabetes [22–24]. The results from previous studies of the anti-inflammatory effects of vitamin D supplementation have been inconsistent. Some studies have found vitamin D supplementation to improve the levels of certain inflammatory markers, such as IL-6, IL-10, CRP, or TNF-*α* [6, 10, 25–27], whereas others have found no effects on any of the studied markers [19, 28–30]. However, only a few studies have been conducted in subjects with prediabetes [6, 19, 31] and, to our knowledge, no study thus far has investigated the impact of vitamin D supplementation on IL-1RA.

Our study was conducted during October to April, the period of low UVB light exposure from the sun in Finland, to allow as unbiased supplemental vitamin D effect as possible. Strengths of the study were the homogenous study population and use of two different, sufficiently large vitamin D_3_ doses, which enabled studying possible dose response. A limitation of the study was the unbalanced gender distribution, especially the low number of females. Another limitation is the lower than planned total number of study subjects, which was due to the need to close recruitment to allow a sufficiently long supplementation during the low UVB exposure period, and the higher than anticipated average 25(OH)D_3_ concentration of the study population at the start of the study. However, the fairly consistent results of our study do not suggest a marked change in observed effects even if some more subjects would have been included, or if* intention-to-treat* analysis had been carried out instead of* per-protocol* analysis, given that* per-protocol* tends to exaggerate the treatment effects, if anything.

## 5. Conclusions

In conclusion, our study does not support the role of winter-time relatively high-dose vitamin D_3_ supplementation as a means to improve glucose homeostasis in a general ageing population with prediabetes but suggests a modest anti-inflammatory effect.

## Figures and Tables

**Table 1:** Characteristics of the study population (*n* = 68) at the start of the supplementation period.

Variable	Mean or median	Standard deviation or interquartile range
Age (years)^a^	65.7	7.0
25(OH)D_3_ (nmol/L)^b^	57.0	11.0
HbA_1c_ (mmol/mol)^b^	40	3
HbA_1c_ (%)^b^	5.8	2.5
Fasting glucose (nmol/L)^a^	6.1	0.7
120 min glucose (nmol/L)^a^	6.1	2.5
Fasting insulin (mU/L)^b^	12.0	6.0
120 min insulin (mU/L)^c^	61.9	62.3
HOMA2 %beta^b^	82.2	24.1
HOMA2 %IS^a^	70.2	39.2
HOMA2 %IR^b^	1.6	0.8
Waist circumference (cm)^b^	106	9
Body mass index (kg/m^2^)^b^	29.4	2.7
Systolic BP (mmHg)^a^	144	30
Diastolic BP (mmHg)^b^	92	10
Serum PTH (pg/ml)^a^	42.6	12.7
Serum calcium (mM)^b^	2.33	0.07
Serum creatinine (*µ*mol/L)^a^	71.7	14.5
Serum ALAT (U/L)^a^	27.5	17.6
Serum GGT (U/L)^a^	29.4	23.7
Serum TSH (mU/L)^a^	2.6	1.7
Serum HDL cholesterol (mmol/L)^b^	1.4	0.4
Serum LDL cholesterol (mmol/L)^b^	3.2	0.9
Serum triglycerides (mmol/L)^a^	1.3	0.7
Serum ApoA1 (g/L)^b,c^	1.6	0.2
Serum ApoB (g/L)^b,d^	1.0	0.2
Serum adiponectin (*µ*g/mL)^a,c^	4.8	3.3
Serum C-reactive protein (mg/L)^a,c^	1.2	1.5
Plasma TNF receptor II (pg/mL)^a^	2240	480
Plasma IL-6 (pg/mL)^a^	1.3	0.8
Plasma IL-1 beta (pg/mL)^a^	0	0
Plasma IL-1 receptor antagonist (pg/mL)^a^	200	95

^a^Median and interquartile range; ^b^mean and standard deviation;^c^ *n* = 66; ^d^ *n* = 65.

25(OH)D_3_: 25-hydroxyvitamin D_3_; ALAT: alanine aminotransferase; Apo: apoprotein; GGT: gamma-glutamyl transferase; HDL: high-density lipoprotein; HOMA: homeostatic modeling assessment; IL: interleukin; IR: insulin resistance; IS: insulin sensitivity; LDL: low-density lipoprotein; PTH: parathyroid hormone; TNF: tumor necrosis factor; TSH: thyroid-stimulating hormone.

**Table 2:** Changes over time in the outcome parameters by supplementation group (*n* = 66).

Parameter	Placebo (*n* = 21)	40 *μ*g/d (*n* = 24)	80 *μ*g/d (*n* = 21)	*P*_K-W_	*P*_M-WU_^d^	*P*_J-T_
Serum 25(OH)D_3_ (nmol/L)^a^	4.1 (17.3)	27.7 (17.2)^c^	45.0 (23.4)^c^	<0.001	<0.001, <0.001	<0.001
Serum PTH (pg/mL)^b^	5.6 (7.4)^c^	−2.6 (10.8)	−6.4 (9.6)^c^	<0.001	0.003, <0.001	<0.001
Serum calcium (mM)^a^	−0.06 (0.08)^c^	−0.04 (0.07)^c^	−0.03 (0.08)	0.367	—	0.082
HbA_1c_ (mmol/mol)^a^	−1.8 (1.2)^c^	−1.2 (2.0)^c^	−1.0 (2.0)^c^	0.130	—	0.024
HbA_1c_ (%)^b^	−0.2 (0.2)^c^	−0.1 (0.2)^c^	−0.1 (0.2)^c^	0.231	—	0.061
Fasting glucose (nmol/L)^a^	−0.1 (0.4)	−0.1 (0.3)^c^	−0.2 (0.5)^c^	0.483	—	0.104
30 min glucose (nmol/L)^a,e^	−0.4 (1.1)	−0.2 (1.0)	−0.3 (1.4)	0.811	—	0.380
120 min glucose (nmol/L)^a^	−0.5 (1.6)	0.7 (1.7)	0.5 (1.8)	0.039	0.011, 0.021	0.021
Fasting insulin (mU/L)^b^	0.3 (6.1)	0.6 (2.0)	−0.1 (5.3)	0.690	—	0.241
30 min insulin (mU/L)^b^	−0.8 (29.6)	−0.8 (35.1)	−7.7 (31.7)	0.142	—	0.030
120 min insulin (mU/L)^b,e^	1.0 (34.7)	10.5 (33.8)	1.4 (60.6)	0.584	—	0.430
HOMA2 %beta^a^	6.2 (24.5)	8.0 (14.5)^c^	6.6 (17.3)	0.605	—	0.300
HOMA2 %IS^a^	−2.9 (28.8)	−6.0 (23.7)	2.0 (20.6)	0.554	—	0.287
HOMA2 %IR^a^	0.13 (0.71)	0.09 (0.33)	0.03 (0.99)	0.714	—	0.243
Insulin sensitivity index^a^	−0.1 (1.2)	−0.4 (1.4)	0.3 (1.5)	0.624	—	0.398
Insulinogenic index^b,e^	1.5 (9.2)	0.1 (8.4)	−2.8 (7.3)^c^	0.306	—	0.063
Waist circumference (cm)^a,e^	0.02 (3.0)	0.1 (2.3)	0.5 (1.9)	0.741	—	0.311
Body mass index (kg/m^2^)^a^	0.25 (0.86)	0.34 (0.55)^c^	0.30 (0.58)^c^	0.973	—	0.404
Serum ALAT (U/L)^b^	0.3 (10.1)	1.6 (8.7)	0.07 (7.5)	0.345	—	0.505
Serum GGT (U/L)^b^	−0.5 (14.2)	0.9 (7.3)	−1.0 (8.6)	0.462	—	0.211
Serum adiponectin (*µ*g/mL)^b^	−0.3 (1.6)	−0.1 (1.7)	−0.1 (1.1)	0.997	—	0.497
Serum C-reactive protein (mg/L)^b^	−0.01 (2.6)	−0.1 (1.4)	−0.6 (1.0)^c^	0.328	—	0.122
Plasma TNF receptor II (pg/mL)^b^	46.5 (387.5)	−15.8 (265.1)	42.5 (312.7)	0.635	—	0.235
Plasma IL-6 (pg/mL)^b^	−0.2 (0.6)	0.04 (0.7)	−0.1 (0.6)	0.371	—	0.380
Plasma IL-1 beta (pg/mL)^b^	na	na	na	(0.775)	—	(0.287)
Plasma IL-1 receptor antagonist (pg/mL)^b^	19.2 (85.1)	2.7 (43.2)	−8.9 (52.6)	0.383	—	0.070

^a^Values are mean (standard deviation).

^b^Values are median (interquartile range).

^c^Wilcoxon Signed Rank test for the change within group, *P*_WSR_ < 0.05.

^d^Mann-Whitney *U* *P* value for the difference is computed in the following order: placebo and 40 *µ*g/d, placebo and 80 *µ*g/d.

^e^ *n* = 65.

25(OH)D_3_: 25-hydroxyvitamin D_3_; ALAT: alanine aminotransferase; GGT: gamma-glutamyl transferase; HOMA: homeostatic modeling assessment; IL: interleukin; IR: insulin resistance; IS: insulin sensitivity; J-T: Jonckheere-Terpstra; K-W: Kruskal-Wallis; M-WU: Mann-Whitney *U*; PTH: parathyroid hormone.
